# COMMD1 Promotes pVHL and O_2_-Independent Proteolysis of HIF-1α via HSP90/70

**DOI:** 10.1371/journal.pone.0007332

**Published:** 2009-10-05

**Authors:** Bart van de Sluis, Arjan J. Groot, Jeroen Vermeulen, Elsken van der Wall, Paul J. van Diest, Cisca Wijmenga, Leo W. Klomp, Marc Vooijs

**Affiliations:** 1 Complex Genetics Section, DBG-Department of Medical Genetics, University Medical Center Utrecht, Utrecht, The Netherlands; 2 Laboratory for Metabolic and Endocrine Diseases, University Medical Center Utrecht, Utrecht, The Netherlands; 3 Department of Pathology, University Medical Center Utrecht, Utrecht, The Netherlands; 4 Department of Internal Medicine, University Medical Center Utrecht, Utrecht, The Netherlands; 5 Department of Pathology and Laboratory Medicine, University Medical Center Groningen, Groningen, The Netherlands; 6 Department of Genetics, University Medical Center Groningen, Groningen, The Netherlands; Fred Hutchinson Cancer Research Center, United States of America

## Abstract

**Background:**

The Copper Metabolism MURR1 Domain containing 1 protein COMMD1 has been associated with copper homeostasis, NF-κB signaling, and sodium transport. Recently, we identified COMMD1 as a novel protein in HIF-1 signaling. Mouse embryos deficient for *Commd1* have increased expression of hypoxia/HIF-regulated genes i.e. *VEGF*, *PGK* and *Bnip3*. Hypoxia-inducible factors (HIFs) are master regulators of oxygen homeostasis, which control angiogenesis, erythropoiesis, glycolysis and cell survival/proliferation under normal and pathologic conditions. Although HIF activity is mainly controlled by ubiquitination and protein degradation by the von Hippel Lindau (pVHL) tumor suppressor gene other mechanisms have recently been identified that regulate HIF signaling independently of pVHL.

**Principal Findings:**

Here we characterized the mechanism by which COMMD1 regulates HIF-1α protein degradation. We show that COMMD1 competes with the chaperone heat shock protein HSP90β for binding to the NH_2_-terminal DNA-binding and heterodimerization domain of HIF-1α to regulate HIF-1α stability together with HSP70. Inhibition of HSP90 activity with 17-Allylamino-17-demethoxygeldanamycin (17-AAG) increased COMMD1-mediated HIF-1α degradation independent of ubiquitin and pVHL.

**Conclusion/Significance:**

These data reveal a novel role for COMMD1 in conjunction with HSP90β/HSP70 in the ubiquitin and O_2_-independent regulation of HIF-1α.

## Introduction

Copper Metabolism MURR1 Domain 1 protein (COMMD1) is the prototype of the COMMD protein family [1]. This family of proteins is highly conserved between eukaryotes, and some members have been identified in protozoa [1], [2]. The COMM proteins are ubiquitously expressed and characterized by their conserved and distinctive domain (COMM domain) located at the carboxy-terminus. The COMMD domain is important for protein-protein interactions, nuclear export and postranslational modifications of COMMD proteins [3]–[5].

To date the best-characterized COMMD protein is COMMD1. We identified COMMD1 (previous called MURR1) to be mutated in dogs with the hepatic copper storage disorder, copper toxicosis [6], [7]. Recent observations suggest that COMMD1 regulates the biliary copper excretion via ATP7B, a P-type ATPase copper transporter that is mutated in patients with the copper storage disorder Wilson's disease [8], [9]. It appears that COMMD1 participates in the protein quality control of ATP7B by mediating the proteolysis of ATP7B [8].

COMMD1 also inhibits basal and cytokine induced NF-κB activity by promoting the ubiquitination and proteasomal degradation of RelA [1], [5], [10]. To understand the *in vivo* role of COMMD1 we generated *Commd1* deficient mice [11]. Remarkably, *Commd1* knockout mice are embryonically lethal, and show abnormal embryonic development, including placental defects. Gene expression analysis in *Commd1* deficient embryos revealed deregulation of the hypoxia inducible factor (HIF) pathway by upregulation of canonical HIF-1 target genes like *Vegfa*, *Pgk1*, *AldoA*, *Bnip3* and others. Conversely, overexpression of COMMD1 resulted in downregulation of HIF-1 activity [11].

HIF-1 is a key factor in oxygen homeostasis. Under conditions of chronic or acute oxygen deprivation (hypoxia), HIF-1 regulates the transcriptional activation of target genes involved in angiogenesis, erythropoiesis, glycolysis and cell survival/proliferation [12]. HIF-1α is a basic-helix-loop-helix (bHLH)-PAS (Per, ARNT, SIM) protein and forms together with HIF-1β the transcription factor HIF-1 [13]. The N-terminal bHLH and PAS domain of HIF-1α and HIF-1β mediates heterodimerization and DNA binding [14]. The cellular HIF-1α protein level is tightly regulated by oxygen. During normoxia HIF-1α is continuously hydroxylated by prolyl hydroxylases (PHD1-3), which, in turn recruits the VCB-Cul2 (von Hippel-Lindau protein (pVHL), Elongin B/C, Cullin2) E3 ubiquitin ligase complex [15]–[18]. VCB-Cul2 ubiquitinates HIF-1α and thereby targets it for 26S proteasomal degradation. HIF-1α is hydroxylated at conserved proline residues (402 and 564) located within the oxygen-dependent degradation domain (ODDD). Under hypoxic conditions, hydroxylase activities of PHDs are attenuated, resulting in HIF-1α stabilization and eventually the formation of active HIF-1 that regulate gene expression of HIF-1 target genes by binding to hypoxia responsive elements (HREs) [14], [19].

Although HIF-1 is generally regulated by pVHL, it has become apparent that more pathways are involved in the regulation of basal HIF-1α levels in an oxygen- and pVHL-independent manner (reviewed in [20], [21]). Recent studies showed that the heatshock protein 90β (HSP90β) binds HIF-1α and is necessary for proper maturation and stabilization of HIF-1α under normoxic and hypoxic conditions. HSP90β is a molecular chaperone that is involved in many different cellular processes and contributes to post-translation maturation and folding of client proteins (reviewed by [22]). HSP90 inhibitors, e.g. geldanamycin or 17-Allylamino-17-demethoxygeldanamycin (17-AAG), both induce ubiquitin-independent and ubiquitin-dependent degradation of HIF-α independent of pVHL [23]–[28].

In this study, we further characterized the role of COMMD1 in HIF-1α proteolysis and demonstrated that COMMD1 regulates HIF-1α stability in a pVHL/O_2_ and ubiquitin-independent manner by competing with HSP90β for binding to HIF-1α. Our findings suggest that COMMD1 is associated with the HSP90β/HSP70 chaperone regulatory axis in regulating HIF-1 pathway activity, and explain why loss of COMMD1 attenuates HIF-1α proteolysis *in vivo*.

## Results

### COMMD1 mediates HIF-1α protein stability

**Figure 1:**
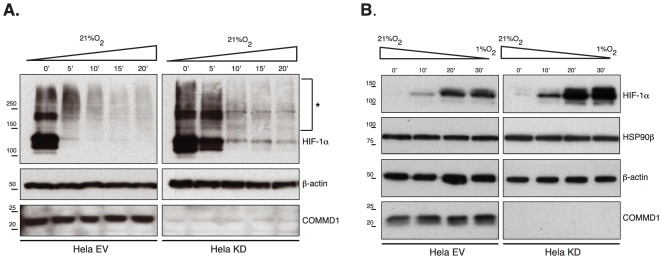
COMMD1 mediates HIF-1α protein stability. (A) The stability of HIF-1α protein was determined in HeLa COMMD1 knockdown (KD) and control (EV) cells by immunoblotting. After hypoxia (1% O_2_) for 8 hours the cells were reoxygenized (21% O_2_) for the indicated time prior to cell lysis. Post-translational modified HIF-1α, can be seen as high-molecular mass-proteins detected by anti-HIF-1α antibody indicated with asterisks. β-actin expression was used as a loading. (B) The maturation/stability of HIF-1α protein, was determined in HeLa COMMD1 knockdown (KD) and control cells (EV) by immunoblotting. After culturing the cells under normoxic conditions (21% O_2_) cells were exposed to 1% O_2_ for the indicated times. HSP90β expression was determined and β-actin expression was used as a loading control. This experiment was repeated three times with similar results.

We previously demonstrated that COMMD1 physically interacts with HIF-1α and that loss of COMMD1 is associated with increased HIF-1α protein stability *in vivo* and *in vitro*. To further define the role of COMMD1 in HIF-1α regulation, we generated stable HeLa COMMD1 knock down cells by transfection of pSuper–shRNA, as described previously [11]. This cell line and a control HeLa cell line were cultured for 18 h at 1% O_2_, which resulted in comparable HIF-1α protein stabilization in both cell lines (Figure 1A). Upon reoxygenation (ambient O_2_), HIF-1α levels decreased in both cell lines but in COMMD1 knockdown cells the rate of HIF-1α degradation was markedly reduced. After 5 min of normoxic exposure, high HIF-1α level was still seen in COMMD1 knockdown cells, whereas HIF-1α was almost undetectable in the control cell line. In both cells lines, higher molecular weight species of HIF-1α were detected by immunoblotting that appeared more abundant in reoxygenated COMMD1-silenced cells. The identity of these higher molecular weight band is unknown at present but may represent posttranslational modification such as ubiquitination. These findings are in agreement with a role for COMMD1 in HIF-1α degradation as observed in HEK 293T cells [11]

Recently we suggested that COMMD1 promotes the proteolysis of newly synthesized and incorrectly folded ATP7B proteins [8]. To investigate whether COMMD1 is also involved in the stability of newly synthesized HIF-1α, we studied the dynamics of HIF-1α maturation at several time-points after oxygen deprivation in control or COMMD1 knockdown cells. These cells, cultured under normoxia (21% O_2_), were exposed to hypoxia (1% O_2_) after removal of the culture medium to obtain rapid deoxygenation. Upon oxygen deprivation, the levels of HIF-1α rapidly increased in both cell lines in a time-dependent fashion (Figure 1B). However, HIF-1α level in COMMD1 silenced cells were markedly higher at all time-points, than in control cells (Figure 1B). Similar results were obtained with silenced COMMD1 HEK 293T cells (data not shown). Altogether, these data indicate that COMMD1 controls the protein degradation and maturation of HIF-1α.

### COMMD1 competes with HSP90β for binding to HIF-1α

**Figure 2:**
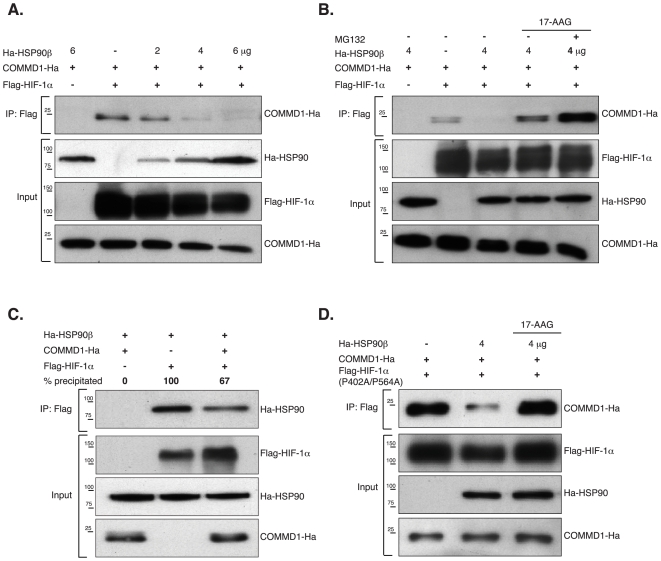
HSP90β competes with COMMD1 for binding to HIF-1α. (A). HEK 293T cells were transfected with cDNA constructs encoding COMMD1-Ha, Flag-HIF-1α, or Ha-HSP90β with increase amounts of plasmid DNA as indicated. Whole cell lysates were immunoprecipitated with anti-Flag antibody and subjected to Western Blot analysis. (B) Anti-Flag immunprecipitations using HEK 293T whole cells lysates expressing Ha-HSP90β, COMMD1-Ha, Flag-HIF-1α. Prior to cell lysis and immunoprecipitation cells were treated with vehicle, 17-AAG (2 µM, 8 hr), or MG132 (5 µM, 8 hr) as indicated. (C) Cells were cotransfected with Ha-HSP90β, COMMD1-Ha, Flag-HIF-1α. Whole cell lysates were subjected to immunprecipation with anti-Flag antibody. Bands were quantified by densitometry using Quantity One (Bio-Rad) to calculate the quantity of the ratio of precipitated HSP90β relative to the input levels. The percentage of precipitated HSP90β is indicated (D) HEK 293T whole cells lysates expressing Ha-HSP90β, COMMD1-Ha, Flag-HIF-1α (P402A/P564A) were used for immunprecipation with anti-Flag antibody. Prior to cell lysis and immunoprecipitation cells were treated with vehicle or 17-AAG (2 µM, 8 hr). All data represent at least two experiments with comparable results.

We recently showed that COMMD1 directly binds to the N-terminal region of HIF-1α and competes with HIF-1β for binding to HIF-1α (van de Sluis et al., submitted). The heat shock protein HSP90β binds also to this region of HIF-1α and is essential for the stability and maturation of HIF-1α protein [25]. Since both proteins bind to same region, we examined the interaction between HIF-1α and COMMD1 in relation to HSP90β levels. COMMD1-Ha and Flag-HIF-1α were transfected in HEK 293T cells in the presence of increasing amounts of Ha-HSP90β. COMMD1-Ha co-immunoprecipitated with HIF-1α under all conditions, but the level of COMMD1-HIF-1α binding gradually decreased with increasing HSP90β levels (Figure 2A). The competition between HSP90β and COMMD1 for HIF binding could be reversed when culturing in the presence of an HSP90 specific inhibitor, 17-Allylamino-17-demethoxygeldanamycin (17-AAG). Moreover, the interaction between COMMD1 and HIF-1α was enhanced after 17-AAG treatment compared to mock treated cells or cells not transfected with HSP90β. This interaction was even more pronounced when proteasomal degradation was blocked (Figure 2B). Consistent with a competition model, we found that overexpression of COMMD1 decreased HSP90β binding to HIF-1α (Figure 2C). To address whether COMMD1/HSP90 mediated HIF-1α degradation was dependent on pVHL we used a hydroxy-prolyl HIF-1α mutant (P402A/P564A), which is not targeted for pVHL-mediated proteasomal degradation. HSP90β also decreased the binding of COMMD1 with the hydroxy-prolyl HIF-1α mutant and was completely abrogated in the presence of 17-AAG (Figure 2D) indicating that the effect of COMMD1 was independent of proly-hydroxylation and pVHL binding to HIF-1α. These results show that the binding of COMMD1 to HIF-1α is in competition with active HSP90β, in a dose-dependent manner.

### COMMD1 promotes HIF-1α degradation in an ubiquitin independent manner

**Figure 3:**
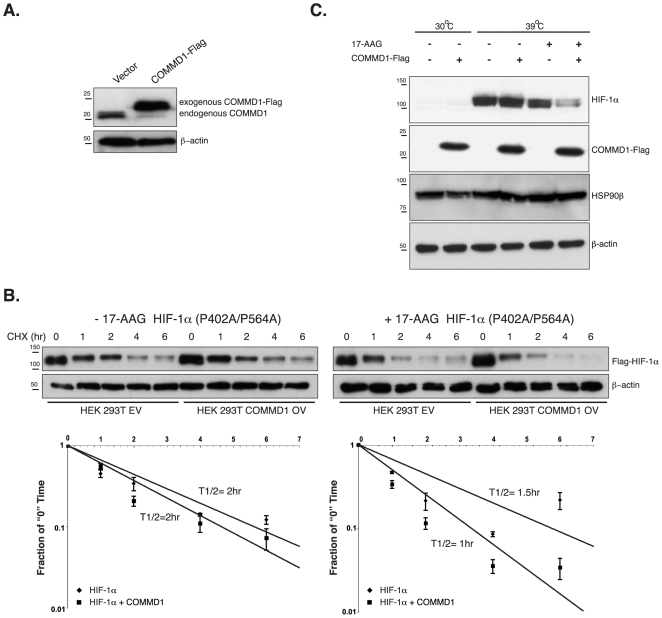
COMMD1 increased HIF-1α proteolysis in an HSP90-dependent but ubiquitin independent manner. HEK 293T stably overexpressing COMMD1-Flag (OV) and control cells (EV) (A) were transfected with Flag-HIF-1α (P402A/P564A). Cells were untreated or treated with 17-AAG (2 µM) and CHX (40 µg/ml) was added to the cells. HIF-1α levels were determined by Western Blot analysis. Bands were quantified by densitometry using Quantity One (Bio-Rad) to calculate the half-life values (T1/2) of HIF-1α (P402A/P564A). For each sample, mean values±SEM are shown (n = 3), significant difference were determined using a Student *t*-test, p = 0.0438 (C) Ts20 cells, containing thermolabile ubiquitin activation enzyme E1, were transfected with empty vector or COMMD1-flag. Cells were cultured under permissive temperature (30°C) or nonpermissive temperature (39°C) for 10 hr. Cells were treated with vehicle or 17-AAG for 8 hr prior to cell lysis and western blot analysis. All data represent three experiments with comparable results.

Since HSP90β regulates the HIF-1α protein stability in a pVHL-independent manner we investigated the stability of the hydroxy-prolyl HIF-1α mutant (P402A/P564A) in HEK 293T cells stably overexpressing COMMD1-Flag (Figure 3A). HEK-293T-COMMD1 cells expressed approximately 3.5-fold higher COMMD1 levels than control cells. The endogenous COMMD1 protein was repressed in HEK293-COMMD1-Flag cells (Figure 3A), a phenomenon also observed and described by others [3]. We expressed Flag-HIF-1α (P402A/P564A) in both cell lines in the presence or absence of 17-AAG. After adding cyclohexamide (CHX), to block new protein synthesis, we assessed whether COMMD1 overexpression resulted in a decrease of HIF-1α stability. No clear difference in HIF-1α stability could be detected between COMMD1 overexpression and control HEK 293T cells in the absence of 17-AAG, whereas the half-life of HIF-1α was markedly reduced in control cells and COMMD1 overexpression cells treated with 17-AAG (Figure 3B). However, inhibition of HSP90β in COMMD1 overexpressing cells significantly decreased the half-life of HIF-1α compared to control cells (Figure 3B). Next, we studied whether COMMD1 promotes the degradation of HIF-1α in an ubiquitin-independent manner by studying the expression of endogenous HIF-1α in a Chinese hamster ovary (CHO) cell line (Ts20), which contains thermolabile ubiquitin activation enzyme E1 [29]. At non-permissive temperature (39°C) the ubiquitin conjugation is dysfunctional and leads to HIF-1α stability in an oxygen-independent manner (Figure 3C). Under these conditions HIF-1α levels were clearly reduced when HSP90β activity was inhibited. Interestingly, overexpression of COMMD1 together with 17-AAG treatment considerably decreased endogenous HIF-1α levels in the absence of ubiquitination (Figure 3C). These data further demonstrate that COMMD1 mediates HIF-1α stability in concert with HSP90β activity in a manner independent pVHL-mediated polyubiquitination.

### COMMD1 forms a tripartite complex with HSP70 and HIF-1α

**Figure 4:**
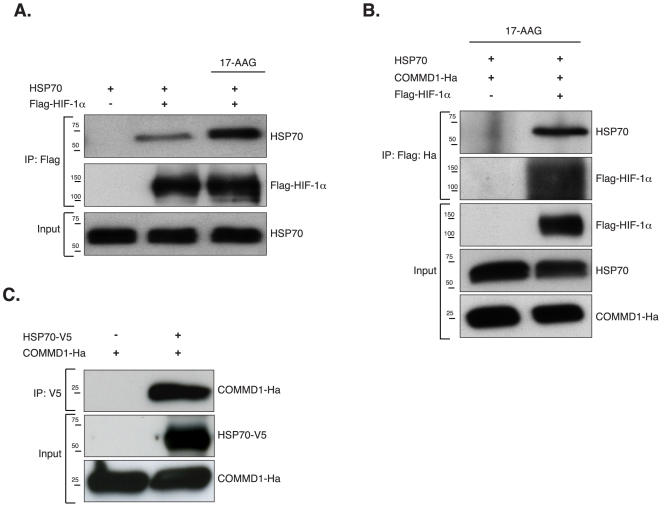
COMMD1 forms a tripartite complex with HSP70 and HIF-1α. (**Α**) HEK 293T whole cells lysates expressing HSP70 and Flag-HIF-1α were used for anti-Flag immunprecipitation. Prior to cell lysis cells were treated with vehicle or 17-AAG (2 µM, 8 hr) as indicated. (B) HEK 293T cells were transfected with plasmids encoding HSP70, COMMD1-Ha and Flag-HIF-1α. Lysates of cells, which were treated with 17-AAG (2 µM, 8 hr), were used for sequential immunoprecipitation. Anti-Flag precipitates were eluted with Flag-peptides and eluates were used for immunoprecipitation with anti-HA antibody. Whole cell lysates, and immunoprecipitates were subjected to Western Blot analysis. (C) Using anti-V5 antibody HSP70 was immunoprecipitated from HEK 293T cell lystates expressing HSP70-V5 and COMMD1-Ha. Whole cell lysates, and precipitates were analyzed by SDS-PAGE and immunoblotting as indicated.

Heat shock protein HSP70 also binds to HIF-1α in an HSP90β-dependent manner and facilitates the proteolysis of misfolded and immature HIF-1α [25] in a manner similar seen for COMMD1 (Figure 2B,D). We therefore hypothesized that COMMD1 may act as a ‘scaffold’ protein involved degradation of HIF-1α together with HSP70. First we studied the interaction between HSP70 and HIF-1α under normal conditions and after attenuating HSP90 activity. Blocking HSP90 clearly increased the binding between HSP70 and HIF-1α (Figure 4A). Second, we investigated whether HSP70 and COMMD1 form a protein complex with HIF-1α. To address this we sequentially precipitated Flag-HIF-1α from cells, in which we co-expressed Flag-HIF-1α, HSP70 and COMMD1-Ha. Transfected cells were treated with 17-AAG to block HSP90β activity prior to cell lysis. Flag-precipitates were eluted with Flag-peptide and subsequently re-precipitated with anti-Ha to enrich for COMMD1 containing immunocomplexes. As demonstrated in Figure 4B, HSP70 could be found in complex with COMMD1 in cells expressing HIF-1α. To delineate whether COMMD1 is associated with HSP70, HSP70 was immunoprecipitated from HEK 293T cell lysates expressing HSP70-V5 and COMMD1-Ha. COMMD1 was clearly present in HSP70-immunoprecipates (Figure 4C). These data indicate that COMMD1 can form with HSP70 a tripartite complex with HIF-1α.

## Discussion

COMMD1 has been recognized as a protein that plays an important role in wide range of biological processes, including copper metabolism, NF-κB signaling and sodium transport (reviewed in [21]). We recently identified COMMD1 as a novel protein regulating HIF-1 signaling as embryos deficient for *Commd1* display upregulation of HIF-1 target genes with corresponding increased HIF-1α protein stability [11]. In this study, we further characterized the mechanism of COMMD1 in HIF-1α regulation and demonstrated that COMMD1 competes with HSP90β for binding to HIF-1α to facilitate HIF-1α degradation. Overexpression of COMMD1 enhances the protein degradation of HIF-1α in an HSP90-dependent but pVHL/O_2_ and ubiquitin-independent manner (Figure 3). Previous studies reported that upon HSP90 inhibition, a protein complex consisting of HSP70 and HIF-1α is formed to facilitate 20S proteasomal degradation in an ubiquitin-independent fashion [25], [30], [31]. Here we showed that COMMD1 is in complex with HSP70/HIF-1α suggesting that COMMD1 acts in concert with HSP70 to facilitate proteolysis of HIF-1α in an HSP90-dependent manner.

The effect of COMMD1 on HIF-1α stability is also evident during the early transitions between hypoxia and normoxia and vice versa. In cells reoxygenation in the absence COMMD1 results in a delay in the rate of HIF-1α degradation (Figure 1A). It is well established that under normal oxygen conditions, HIF-1α levels are downregulated by polyubiquitination and proteosomal degradation (18). We therefore hypothesize that the higher molecular weight protein, detected by the HIF-1α antibody, represents polyubiquitinated-HIF-1α species. We have previously observed the same HIF-1α pattern in HEK 293T COMMD1 deficient cells upon reoxygenation ([11]). The persistence of polyubiquitinated HIF-1α in COMMD1 deficient cells may suggest that COMMD1 does not affect HIF-1α polyubiquitination per se but assists the proteasomal degradation of HIF-1α. This could also explain why HIF-1 transcriptional activity is higher in the absence of COMMD1 (11). Together with our observation that COMMD1 interacts with the 20S proteasome (supplemental Figure S1) we hypothesize that COMMD1 may escort HIF-1α protein to the proteasome. Furthermore, the increased interaction between COMMD1 and 20S after blocking proteasomal degradation, supports a more general role for COMMD1 in the 20S proteasome. Ubiquitin-independent degradation of HIF-1α directly by the 20S proteosome has also been reported by others and seems to be involved in regulating constitutive degradation of HIF-1α during hypoxia [31]. While HIF-1α can interact with the 20S proteasome subunit PMSA7 (α-4 subunit), it is unknown yet whether this is a direct process or regulated by other proteins [32]. Although more studies are needed, these data shed light on a novel pathway of HIF-1α regulation by the 20S proteasome in an O_2_ and ubiquitin-independent manner.

A competition with HSP90β for binding to HIF-1α was also reported for Receptor for activated protein kinase C 1 (RACK1) [26]. Similar as for COMMD1, RACK1 reduces HIF-1 transcriptional activity and mediates HIF-1α protein degradation independent of O_2_-and pVHL. However, in constrast to COMMD1, RACK1 recruits the Elongin-C/B ubiquitin ligase complex and regulates HIF-1α stability in an ubiquitin-dependent fashion. Importantly, we found no effect of COMMD1 on RACK1-HIF-1α binding (supplemental Figure S2) suggesting that COMMD1 does not compete with RACK1 for binding to HIF-1α and regulates HIF in a distinct manner.

Interestingly, COMMD1 also promotes the proteolysis of the copper transporting p-type ATPase ATP7B [8]. This study established a stronger binding between COMMD1 and ATP7B mutants that are associated with mis-localization and protein instability compared to wild-type ATP7B. It has been postulated that COMMD1 is engaged in the quality control and protein folding of newly synthesized ATP7B. Although recent data demonstrated interaction between ATP7B and the molecular chaperones HSP70 and HSP90β (unpublished data, P. van den Berghe and L.W. Klomp) the mechanism of COMMD1 in regulating ATP7B degradation is largely unknown. Further studies are needed to reveal whether COMMD1 also cooperates with HSP70 and HSP90β to regulate the structural integrity of ATP7B by assisting the proteolysis of misfolded ATP7B proteins

**Figure 5:**
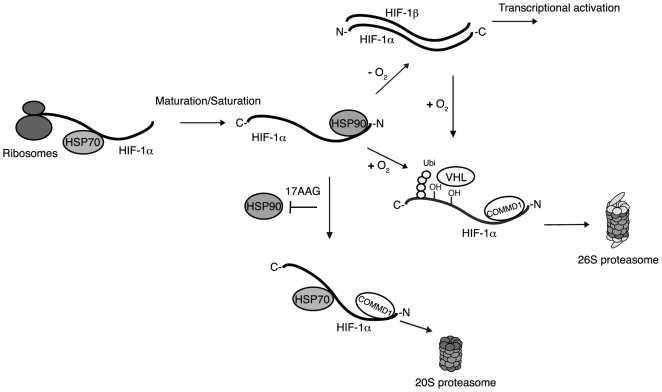
Schematic representation of the mechanism of COMMD1-associated HIF-1α degradation. Under normoxic conditions, newly synthesized HIF-1α binds to HSP70 and HSP90 for proper maturation, but it is immediately degraded by the 26S proteasome after it is hydroxylated and ubiquitinated in a pVHL dependent process. Under hypoxic conditions, HIF-1α is not subjected to hydroxyl- and ubiquitin-dependent degradation and heterodimerizes with HIF-1β, and binds to hypoxia response element (HRE) containing promoters in target genes. Upon reoxygenation HIF-1α is ubiquitinated by pVHL, and COMMD1 is bound to HIF-1α to facilitate proteasomal degradation. Inactivation of HSP90 (e.g. using 17-AAG or Geldanamycin) leads to incorrect folding of HIF-1α and the formation of a heterocomplex with HSP70 and COMMD1, and subsequently degradation by the 20S proteasome. Altogether, in this study we suggest that COMMD1 facilitates the proteasomal degradation of HIF-1α after reoxygenation or HSP90 inhibition independent of posttranslational modification by ubiquitin.

Taken together, this study demonstrates that COMMD1, in complex with HSP70, facilitates the proteasomal degradation of HIF-1α in an HSP90-dependent and ubiquitin independent manner (Figure 5). In addition, these findings highlight a potentially novel role of COMMD1 protein in escorting client proteins to the proteasome. Importantly, hypoxia is a hallmark of solid cancers and HIF-1 activity correlates with a poor prognosis in many cancers [33], [34]. Currently, HSP90 inhibitiors (e.g. 17-AAG) are being tested in clinical trials as potential anti-cancer agents [35]. Therefore, further elucidation of the molecular mechanism of HSP90-dependent proteolysis of proteins may reveal additional opportunities for modulation of HIF-1 activity in human disease, including cancer.

## Materials and Methods

### Construction of expression constructs

Flag-HIF-1α construct was previously described [11]. Mutants for full length HIF-1α P402A/P564A were generated using the QuickChange® XL Site directed Mutagenesis Kit (Stratagene, La Jolla, CA, USA) according to the manufacturer's protocol, using the primers P402A forward 5′-CTTTAACTTTGCTGGCCGCAGCCGCTGGAGACAC-3′ and P402A reverse 5′-GTGTCTCCAGCGGCTGCGGCCAGCAAAGTTAAAG-3′ and P564A forward 5′-CTTGGAGATGTTAGCTGCCTATATCCCAATGGATG-3′ and P564A reverse 5-CATCCATTGGGATATAGGCAGCTAACATCTCCAAG-3′. pcDNA3.1-Ha-HSP90β was kindly provided by Dr. D. Manor (Case Western Reserve University, Cleveland, OH, USA), pcDNA3.1-HSP70 and pcDNA5/FRT/TO-HSP70-V5 was kindly provided by Dr. H. Kampinga (Department of Cell Biology, UMC Groningen, the Netherlands). A plasmid containing the RACK1 cDNA was kindly provided by Dr. D. Qian and recloned in p3xFLAG-CMV10.

### Cell culture, transfections, co-immunoprecipitation, and immunoblotting

HEK 293T and HeLa cells were cultured in Dulbecco's modified Eagle medium supplemented with 10% fetal bovine serum, penicillin-streptomycin, L-glutamine. Cells were cultured at 5% CO_2_, 21% O_2_ for normoxia and 1% O_2_ for hypoxia in an Invivo_2_ Hypoxia Workstation 1000 (Biotrace International, UK) at 37°C. Stable HeLa COMMD1 knockdown cells were generated as previously described [11]. Monoclonal were selected by culturing on a medium supplemented with 1 µg/ml puromycin (Sigma-Aldrich, Zwijndrecht, the Netherlands). Ts20 cells were cultured in MEM Alpha medium+GlutaMAX, supplemented with 10% fetal bovine serum, penicillin-streptomycin. Ts20 cells were cultured at 5% CO_2_, 21% O_2_ at 30°C.

Linear polyethylenimine (P-PEI, Polysciences Inc, Warrington, PA, USA) was used for tranfections. After transfection, cells were lysed in lysis buffer (0.4 M NaCl, 0.1% NP-40, 10 mM Tris-HCl (pH 8.0), 1 mM EDTA) supplemented with 1 mM Na_3_VO_4_, protease inhibitors (Roche, Basle, Switzerland), and 10 mM dithiothreitol. In several experiments MG132 (5 µM, 8 hr) was used to prevent proteasomal degradation of HIF-1α and to attain constant HIF-1α levels. HSP70-V5 expression was achieved by adding doxycycline after transfection. Cell line extracts representing 30 µg protein were transferred onto Hybond-P membranes as explained previously [11]. Immunoblots were probed with anti-COMMD1 antiserum (1∶5,000) [6], anti-Flag (F1804; Sigma-Aldrich), anti-Ha (clone HA-7, Sigma-Aldrich), anti-β-actin monoclonal antibodies (clone AC-74; Sigma-Aldrich), anti-HIF-1α (clone 54; BD Bioscience, Alphen aan de Rijn, the Netherlands), anti-HSP70 (sc-1060, Santa Cruz), anti-HSP90β (SPA-843, Stressgen, Victoria, British Columbia, Canada).

## Supporting Information

Figure S1 COMMD1 interacts with 20S proteasome. Glutathione-sepharose precipitations using cell lysates of HEK 293T transfected with GST or COMMD1-GST. Prior cell lysis, cells were untreated (mock treated, DMSO) or treated with MG132 (5 µM, 8 hr) as indicated. Proteins were detected with anti-GST or anti-20S α-subunit (α1, 2 ,3 ,5 ,6 & 7, Biomol, clone MCP231) as indicated. Input designates direct analysis of cell lysates.
(0.34 MB PDF)

Figure S2 COMMD1 does not compete with RACK1 for binding to the N-terminus of HIF-1α. Glutathione-sepharose precipitations using cell lysates of HEK 293T transfected with GST or GST-HIF-1α (1–300), RACK1-Flag or COMMD1-Ha with increased amounts of plasmid as indicated. Precipitates were washed and separated by SDS-PAGE and immunoblotted as indicated. Input designates direct analysis of cell lysates.
(5.03 MB PDF)
