# Supplementary figures and images for: COMMD1 Promotes pVHL and O2-Independent Proteolysis of HIF-1α via HSP90/70
Source: PLoS One. 2009 Oct 5;4(10):e7332. doi: 10.1371/journal.pone.0007332 (PMC2750754; doi:10.1371/journal.pone.0007332)

Supplementary Figure S1  
van de Sluis et al

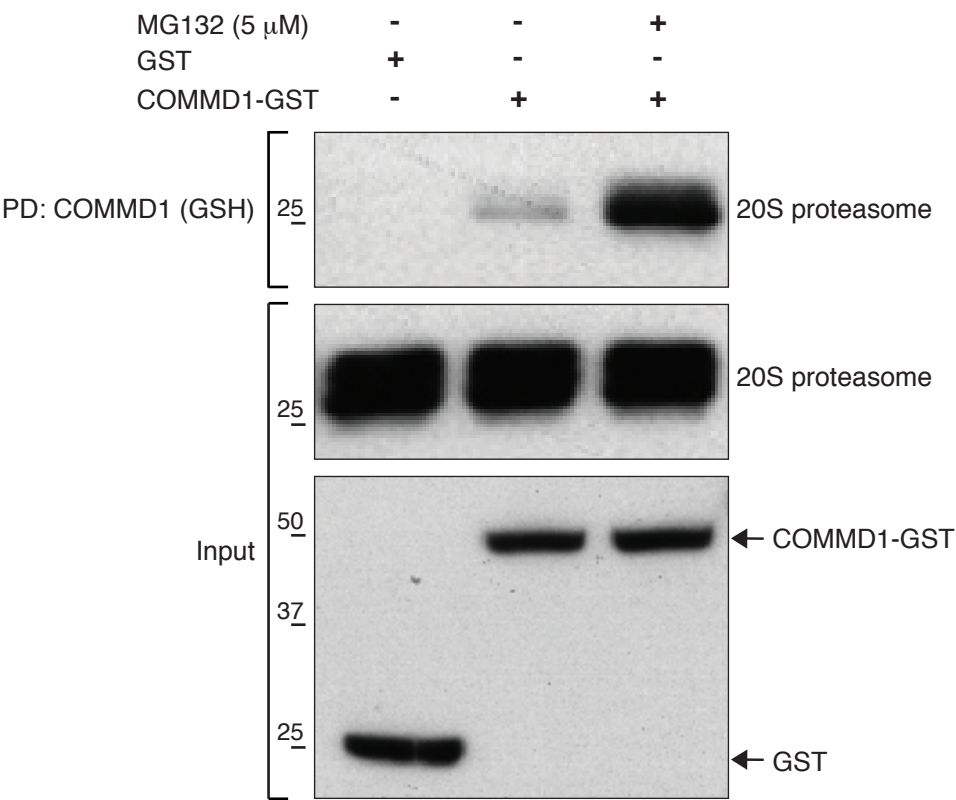

Supplement: Figure S1 — COMMD1 interacts with 20S proteasome. Glutathione-sepharose precipitations using cell lysates of HEK 293T transfected with GST or COMMD1-GST. Prior cell lysis, cells were untreated (mock treated, DMSO) or treated with MG132 (5 µM, 8 hr) as indicated. Proteins were detected with anti-GST or anti-20S α-subunit (α1, 2 ,3 ,5 ,6 & 7, Biomol, clone MCP231) as indicated. Input designates direct analysis of cell lysates. (0.34 MB PDF) [file pone.0007332.s001.pdf]

Supplementary Figure S2  
van de Sluis et al

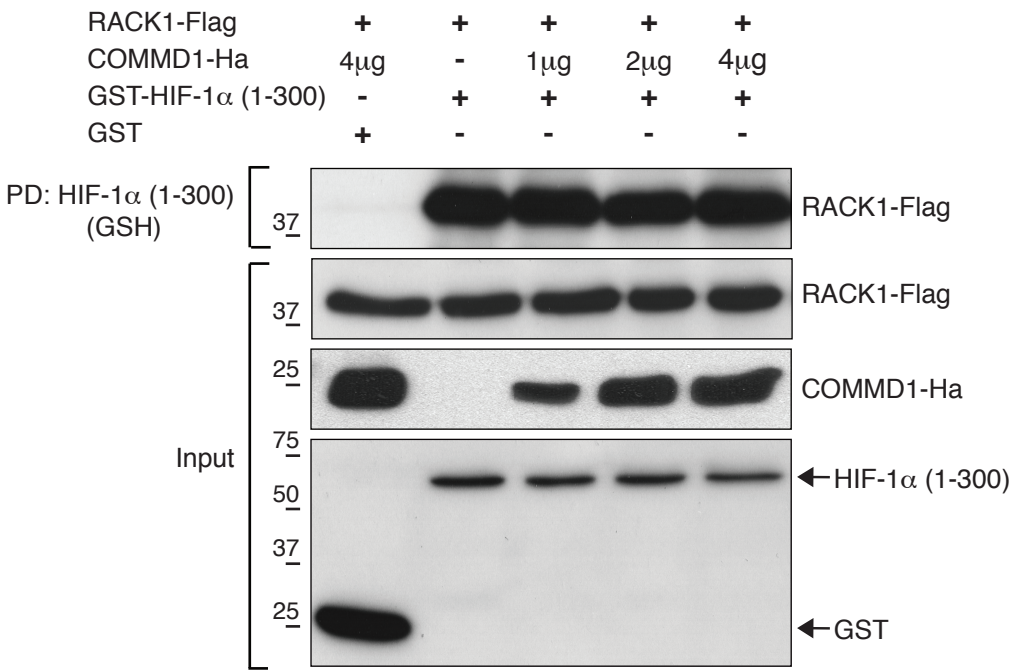

Supplement: Figure S2 — COMMD1 does not compete with RACK1 for binding to the N-terminus of HIF-1α. Glutathione-sepharose precipitations using cell lysates of HEK 293T transfected with GST or GST-HIF-1α (1–300), RACK1-Flag or COMMD1-Ha with increased amounts of plasmid as indicated. Precipitates were washed and separated by SDS-PAGE and immunoblotted as indicated. Input designates direct analysis of cell lysates. (5.03 MB PDF) [file pone.0007332.s002.pdf]
